# Assessment of the Child-Pugh Score, Model for End-Stage Liver Disease Score, Fibrosis-4 Index, and AST to Platelet Ratio Index as Non-endoscopic Predictors of the Presence of Esophageal Varices and Variceal Bleeding in Chronic Liver Disease Patients

**DOI:** 10.7759/cureus.73768

**Published:** 2024-11-15

**Authors:** Suha Tarannum, Taneem Ilyas, Sumaiya Tarannum Shaik, Noorin Sultana, Md Nuzhath Saniya, Amulya Madhulika Mynampati, Kukunoor Akshatha Nayak, Sowmya Gogikar, Ramesh Kumar

**Affiliations:** 1 Internal Medicine, Osmania Medical College, Hyderabad, IND; 2 Medicine, Osmania Medical College, Hyderabad, IND; 3 Gastroenterology and Hepatology, Osmania General Hospital, Hyderabad, IND

**Keywords:** aspartate aminotransferase-to-platelet ratio index (apri score), child-pugh classification, child-pugh score, esophageal varices high risk varices, fibrosis-4 (fib-4) score, gastroesophageal varices, meld-na, model for end stage liver disease (meld)

## Abstract

Background

Esophageal varices (EVs) develop as a complication of chronic liver disease and, when left unaddressed, can lead to variceal hemorrhage manifesting as severe hematemesis and occasionally, melena. Due to its frequent negative associations, early diagnosis and the implementation of non-selective beta blocker primary prophylaxis are imperative. Although upper gastrointestinal endoscopy has historically been used to image and identify EVs, patients frequently find this intrusive treatment to be uncomfortable and burdensome. It can also be expensive and challenging for patients who live in remote places and healthcare deserts, where access to healthcare is limited. Therefore, it is crucial to identify non-invasive markers for the prediction of variceal bleeding and EVs in individuals with chronic liver disease.

Methodology

A cross-sectional observational study was done at Osmania General Hospital, a tertiary healthcare center in Hyderabad, India. The study sample consisted of patients with chronic liver disease who underwent upper gastrointestinal endoscopy during the study period in keeping with the inclusion and exclusion criteria. In a sample of 103 patients, the mean age was 10.72±45.55 years, with 22 females (21.4%) and 81 males (78.6%). The majority (85, 82.5%) had alcoholic chronic liver disease, while 14 (13.6%) had other etiologies, and four (3.9%) had infectious causes. Data were collected to calculate the Child-Pugh score, AST to platelet ratio index (APRI), model for end-stage liver disease (MELD) score, and fibrosis-4 (FIB-4) index. The patients were observed and followed up for a duration of three months. The data were evaluated using chi-squared tests and independent t-tests, chosen according to their relevance, to assess the utility of these scores as non-endoscopic predictors of EVs and esophageal variceal bleeding (EVB).

Results

The results indicated that only the FIB-4 index was found to be a significant predictor of Grade 2 or higher grades of EV according to the Pacquet classification. The FIB-4 index was significantly higher in the Grade 2 or higher EV group (p = 0.029) with t(101) = 1.98.

Conclusion

Thus, the study demonstrates that ≥ Grade 2 EV on upper gastrointestinal endoscopy can be predicted using the FIB-4 index. Even though our study shows that the FIB-4 index is a useful noninvasive predictor of EV, large-scale studies with bigger sample sizes and longer follow-up times are necessary to ensure accurate clinical application.

## Introduction

Esophageal variceal ruptures are associated with a high mortality rate, with around 30% of cases resulting in death due to hemorrhage. Because variceal bleeding has a poor prognosis, it is vital to identify high-risk individuals and prevent a first bleeding episode. Studies of primary prophylaxis with non-selective beta-blockers have shown an established reduction in bleeding risk by 50% to around 15% for large esophageal varices (EVs); hence, early detection of varices prior to the first bleed is crucial. According to current guidelines, endoscopic screening for varices should be performed on all cirrhotic patients [1].

For high-risk patients, endoscopic variceal ligation is a preventive measure that can be used in addition to esophagogastroduodenoscopy (EGD) for the identification and measurement of esophageal varices. One of the universal classifications used in grading EV based on its size and degree of severity is the Pacquet classification. According to Pacquet’s classification, Grade I refers to the presence of micro-capillaries located in the distal esophagus or at the esophagogastric junction. Grade II involves one or two small varices in the distal esophagus. Grade III is characterized by medium-sized varices, regardless of the number. In Grade IV, large-sized varices can be found in any part of the esophagus [2]. Nevertheless, there are high costs, difficulties, and contraindications associated with using endoscopic variceal ligation and screening upper endoscopy to detect and treat esophageal varices. Because endoscopy is invasive, the patient must be well-prepared and have access to the technical expertise required. The patient may encounter difficulties in adhering to the instructions related to the periprocedural period. Consequently, employing non-invasive techniques to detect esophageal varices is likely to avoid some endoscopy-related difficulties and be well-received by patients. Certain patients with cirrhosis may be able to forego invasive screening upper endoscopy if non-invasive testing is able to effectively detect bleeding from EVs [3].

The likelihood of developing EVs can be predicted using non-invasive markers derived from clinical, laboratory, and ultrasonographic characteristics [4]. A number of non-invasive markers have recently been shown to be a straightforward, non-invasive, and more practical alternative to predict the presence of EVs and the risk of variceal bleeds in cirrhotic patients. These markers include the Child-Pugh score, the model for end-stage liver disease (MELD), the AST to platelet ratio index (APRI), fibrosis-4-(FIB-4) index, fibrosis index (FI), etc. However, the conclusions of these prior studies are debatable, and their relevance in clinical practice is questionable. The findings of these studies vary for various liver cirrhosis etiologies and populations [5].

The Child-Pugh scoring system, sometimes referred to as the Child-Pugh-Turcotte score, was created to foresee mortality in individuals with cirrhosis. The idea of predicting the surgical risk for patients undergoing portosystemic shunt surgery for variceal hemorrhage was initially put out. The original idea behind it was to help determine which patients would benefit from elective portal decompression surgery by classifying them into three groups: A: good hepatic function; B: moderately impaired hepatic function; and C: advanced hepatic dysfunction. Five clinical and laboratory criteria, namely, serum bilirubin, serum albumin, ascites, neurological disease, and clinical nutrition status, were employed in their initial scoring system to classify patients. Eventually, Pugh et al. changed the scoring system to replace clinical nutrition status with prothrombin time (PT) [6]. In clinical care, the Child-Pugh score is frequently used to evaluate the severity of liver dysfunction [5]. It has been established that the MELD score is a reliable indicator of survival for patients with acute liver failure, cirrhosis, alcoholic hepatitis, and acute hepatitis. A patient's likelihood of surviving their illness over the next three months is estimated by their MELD score. When it comes to liver transplant cases, the MELD score is also utilized to allocate organs [7]. It can be a useful and objective marker for risk assessment and therapeutic management in patients who have had variceal hemorrhage [8].

One of the most accurate and helpful indicators of the severity of the liver is the FIB-4 score. According to recent research, the FIB-4 index can help patients with nonalcoholic fatty liver disease (NAFLD) predict liver-related events and severe adverse cardiovascular events [9]. The FIB-4 index for liver fibrosis is a noninvasive measure of liver scarring used to determine if a biopsy is necessary in patients with hepatitis C and hepatitis B [10]. The APRI, or aspartate aminotransferase-to-platelet ratio index, is a widely accessible and reasonably priced non-invasive diagnostic method that shows potential to replace liver biopsy in the detection of hepatic fibrosis [11].

There have only been a few studies with large enough sample sizes to evaluate the utility of clinical and laboratory test-based ratings as non-endoscopic predictors for the existence of esophageal varices and the risk of variceal bleeding. Our study aims to evaluate these scores for usage as non-endoscopic predictors of esophageal varices and as predictors of risk of bleeding from esophageal varices in three months for patients with chronic liver disease in South India. Our study is based in a tertiary healthcare center in Hyderabad, India. An additional goal was to compare how well different scores predicted variceal bleeding at the three-month follow-up.

## Materials and methods

Osmania General Hospital, a tertiary healthcare facility in Hyderabad, India, served as the site of this cross-sectional observational study. The study selected participants using a random simple sampling technique, with the goal of including adult patients (over the age of 18) diagnosed with chronic liver disease during the study period. These participants were identified from the outpatient clinics of the Department of General Medicine and the Department of Gastroenterology. The presence of two or more of the following criteria was used to identify chronic liver disease: impaired liver function tests indicative of cirrhosis (elevated liver enzymes, increased international normalized ratio (INR), and decreased serum albumin); or ultrasound findings demonstrating characteristic features of cirrhosis, such as a blunt liver edge, a shrunken or nodular liver with distorted architecture, and associated conditions like splenomegaly or ascites. Patients were limited to those who underwent gastrointestinal endoscopy and gave informed consent.

Patients who were under the age of 18, those who did not give informed consent, those who were hemodynamically unstable, and those who had active hematemesis were all excluded. Furthermore excluded were patients with a history of prior gastrointestinal surgery, such as splenectomy and portosystemic shunt surgery, liver metastasis, hepatocellular carcinoma, extra-hepatic malignancy, portal vein, hepatic vein, or splenic vein thrombosis, and acute or chronic liver failure. Individuals having a history of variceal hemorrhage treated with beta-blockers or endoscopic procedures were also excluded. The precise inclusion and exclusion criteria are described in Table 1.

**Table 1:** Inclusion and exclusion criteria of the study PT/INR: prothrombin time and international normalized ratio

Inclusion criteria	Exclusion criteria
Adult patients (>18 years of age)	Pediatric patients (<18 years of age)
Diagnosis of chronic liver disease	Hemodynamically unstable/acute on chronic liver failure/ Fulminant hepatic failure
Upper gastrointestinal endoscopy performed	History of previous gastrointestinal surgery including splenectomy and portosystemic shunt surgery
Informed consent for the study obtained	History of liver metastasis, hepatocellular carcinoma, or extrahepatic malignancy
	History of endoscopic intervention or beta-blocker therapy as primary prophylactic treatment for varices
	History of portal vein, hepatic vein, and splenic vein thrombosis
	Informed consent for the study not obtained

Obtaining acceptance from the institutional ethical committee (approval number: TEC/OMC/2024/M.No.(8)/Acad-14), the heads of the general medicine and gastroenterology departments, and the principal of Osmania Medical College were among the ethical requirements. The patients themselves provided informed consent, preserving study confidentiality.

Data collection

Each included patient had a thorough history and clinical examination as part of the data collection process. Liver function tests (serum bilirubin, albumin, globulin, PT, and INR) were among the laboratory studies carried out. All patients also had their APRI, FIB-4 score, and MELD score computed. The Child-Pugh classification, which is based on a composite score derived from parameters like encephalopathy, ascites, bilirubin, albumin, and PT-INR, was also used to categorize the severity of liver disease further. The classes are as follows: Class A (total score of five to six), Class B (total score of seven to nine), and Class C (total score of 10 or higher). After laboratory testing for the determination of non-invasive parameters, the results of each patient's upper gastrointestinal endoscopy performed during that visit were documented. After a three-month period, patients were contacted to inquire about the occurrence of variceal hemorrhage. Any episode of hematemesis, melena, or both was indicative of the presence of variceal hemorrhage.

Statistical analysis

Microsoft Excel (Microsoft Corp., Redmond, WA, USA) was used to compile the data, and the IBM SPSS Statistics software, version 24 (IBM Corp., Armonk, NY, USA) was used for analysis. The presence or absence of esophageal varices seen during upper gastrointestinal endoscopy was associated with different ratings. The chi-squared test was used to determine whether the proportions differed from each other in the qualitative data, which was expressed as percentages. The mean and standard deviations were used to express the quantitative data. The independent t-test was used to compare the two means of normal data. The correlation between these scores and the grades of varices based on Pacquet’s classification was analyzed in order to determine the most useful score, or set of scores, for predicting the presence of EVs.

## Results

The study included 103 individuals with chronic liver disease. The mean age was 10.72±45.55 years. Of them, 22 (21.4%) were female and 81 (78.6%) were male patients. Four (3.9%) patients had an infectious etiology, 14 (13.6%) had neither an alcoholic nor an infectious etiology, and 85 (82.5%) had chronic liver disease related to an alcoholic etiology. The Child-Pugh Class C had the greatest number of patients, totaling 58 (56.3%), followed by the Child-Pugh Class B with 35 (34%), and the Child-Pugh Class A with 10 (9.7%). Table 2 displays the distribution of patients among the esophageal varices grades based on Pacquet’s classification.

**Table 2:** Distribution of patients among the grades of esophageal varices based on Pacquet’s classification

Grade of esophageal varices	Frequency (n)	Percentage (%)
0	8	7.8
1	10	9.7
2	64	62.1
3	2	1.9
4	19	18.4
Total	103	100.0

Using an independent t-test to assess non-invasive markers, the Grade 2 or above EV group showed a significantly higher FIB-4 score (p = 0.029). It displayed a t-value of 1.98 along with 101 degrees of freedom. For those with Grade 2 variations or above, the mean FIB-4 score was 8.06±7.87. Those without varices or those with Grade 1 varices on endoscopy had mean FIB-4 scores of 4.33±2.52.

Using an independent t-test, non-invasive indicators like the MELD score and APRI were not shown to be significant. The association between the Child-Pugh class and EV was examined using the chi-square test of independence, and the results showed that the relationship was not significant enough to be used as an EV predictor.

Follow-up was systematically conducted by contacting patients via telephone. All patients were successfully reached, with no instances of loss to follow-up. 76 (73.7%) of the patients experienced EVB during the follow-up. The Child-Pugh score, MELD, APRI, or FIB-4 index were not shown to be relevant in predicting esophageal variceal bleeding (EVB) in three months when non-invasive indicators were evaluated. With a Spearman-Pearson correlation coefficient of 0.892, the FIB-4 index and APRI exhibited strong connections, although APRI was not determined to be a meaningful indicator of EV. This suggests that in order to fully understand the function of these non-invasive markers as non-endoscopic predictors of EV and EVB, bigger prospective studies are required.

## Discussion

Patients with cirrhosis should be very concerned about EV caused by portal hypertension due to the increased risk of bleeding and death that may follow. Because variceal hemorrhage has a bad prognosis, it is vital to identify high-risk individuals and avoid bleeding episodes [4]. Since primary prophylaxis with non-selective beta-blockers has clearly demonstrated a reduction in bleeding risk by 50% to around 15% for large EVs, early detection of varices prior to the first bleed is critical. Guidelines suggest routine endoscopic surveillance of cirrhotic patients for long-term follow-up in order to detect the EV's development and to start preventative steps to stop the EVB when they become large. Because endoscopies are intrusive and pricey, screening with them on a yearly or biannual basis may cause discomfort for the patient. Studies on the use of noninvasive techniques to assess the presence of EV in cirrhotic patients are now being conducted as a result [1]. Since a number of serum indicators and imaging techniques have been shown to correlate favorably with the degree of fibrosis and often take the role of liver biopsy, non-invasive hepatology has emerged as an essential goal. Many of these techniques have been used for non-invasive evaluation of the existence of EV because of the intimate association that exists between liver fibrosis, portal hypertension, and EV [5]. For communities living in rural areas, where access to healthcare may be limited, this study may be of special significance [4].

The outcomes of comparable research done on various groups have been very inconsistent. According to Li et al., the Child-Pugh score was more useful in China as a screening tool to exclude high-risk variants in cirrhosis patients [3]. Gomaa et al. in Egypt discovered that the group with EV had significantly higher ALBI, MELD, and Child-Pugh scores than the group without EV. Additionally, a favorable association was observed between MELD scores and EV grades [5]. This was consistent with the findings of Soga et al., who discovered that patients with EVB had considerably higher Child-Pugh and MELD scores. The MELD score is a noninvasive varices predictor [12]. Similarly, Benedeto-Stojanov et al. found that the MELD score is a good predictor of survival in cirrhotic patients since it was significantly greater in those who passed away from EVB [13]. Increased MELD scores are suggestive of more severe liver disease and may indicate the existence of EVB, according to research by Xu et al. This prospective study's results also showed that a more dynamic profile of disease severity that may be suggestive of cirrhotic consequences can be obtained by tracking changes in the MELD score over time [14].

In our South Indian study on chronic liver disease patients, we found that, according to the Pacquet classification, the presence of EV of Grade 2 or higher was associated with elevated FIB-4 scores, regardless of the underlying etiology. Kothari et al. similarly discovered that the FIB-4 index is among the most reliable non-invasive markers for EV and EVB in alcoholic liver cirrhosis. It can be used for EV screening, risk assessment, EV prediction, and appropriate referral to higher centers [4]. Most importantly, according to Kumar et al.'s findings, the FIB-4 index seems to be the most effective non-invasive marker of liver fibrosis that can be used to screen patients for cirrhosis early on. Among the non-endoscopic techniques examined, only the FIB-4 and MELD scores showed a significant correlation with the existence of EV and EVB [15].

The diagnostic accuracy of the APRI and transient elastography in determining the incidence of EVB was demonstrated to be moderate by Kumar et al. According to this study, noninvasive measures can be used to predict the existence of EV and EVB in addition to endoscopy [16].

Numerous studies have concluded that a combination of scores may be a more reliable predictor than a single indicator. The accuracy, sensitivity, and specificity of using a single clinical marker to predict EV hemorrhage may be limited; however, the diagnostic accuracy may be enhanced by combining many clinical markers. After more research, Li et al. propose that a composite score for the prediction of variceal hemorrhage might be created by combining the albumin-to-bilirubin (ALB), total bilirubin (TBIL), Golgi protein 73 (GP73), and Child-Pugh scores as a single index [3]. The creation of new scores could be taken into consideration akin to EVendo scores. To investigate the precise usefulness of these non-invasive markers for EV and/or EVB prediction, further research involving sizable populations, long-term follow-up, and timely repetition of non-invasive markers may be planned. 

Using a machine-learning algorithm, a new scoring formula called the EVendo score was recently created to detect characteristics strongly linked with EV and varices needing treatment (VNT). Hemoglobin (Hgb), platelet counts, blood urea nitrogen (BUN), ascites presence, INR, and AST level are the easily accessible laboratory tests that comprise the formula. According to Alswat et al., patients with EV had higher APRI, FIB-4, and EVendo scores and significantly decreased platelet counts and ALB. According to their data analysis, the EVendo score performs exceptionally well and consistently when used as a screening tool for EV and VNT in patients with cirrhosis. But to compare the EVendo score with other noninvasive instruments and confirm its effectiveness, a large prospective study could be needed [17].

While our study provides valuable insights, its limitations should be acknowledged. These include potential inter-observer variability in the assessment of varices, which could have impacted the consistency of the findings. Furthermore, extending the follow-up period and increasing the sample size may enhance the precision and reliability of the results. Additionally, biases related to sample selection, measurement inconsistencies, and unaccounted confounding factors may affect the generalizability of the conclusions. Despite these limitations, the study offers promising results that could inform future research, and the findings suggest valuable directions for further exploration.

## Conclusions

In conclusion, while our study reveals that the FIB-4 index is a credible indication of Grade 2 or higher varices on endoscopy, more studies with larger sample sizes are needed to examine and draw conclusions based on a combination of non-invasive procedures and laboratory values. Patients who do not have proper access to healthcare can benefit greatly from new scores designed specifically for EV and EVB prediction. These scores can also expedite the process of choosing patients for esophagogastroduodenoscopy. With enough testing in larger populations, novel scores like EVendo scores show potential and can be used as a non-endoscopic marker and predictor of EV and EVB. The inter-observer heterogeneity in varices assessment is one of our study's limitations. Furthermore, a longer follow-up period with a bigger sample size can yield more precise results.
